# Gay and Bisexual Men’s Perceptions of the Donation and Use of Human Biological Samples for Research: A Qualitative Study

**DOI:** 10.1371/journal.pone.0129924

**Published:** 2015-06-08

**Authors:** Chris Patterson, Lisa M. McDaid, Shona Hilton

**Affiliations:** MRC/CSO Social and Public Health Sciences Unit, University of Glasgow, Glasgow, United Kingdom; Public Health Agency of Barcelona, SPAIN

## Abstract

Human biological samples (biosamples) are increasingly important in diagnosing, treating and measuring the prevalence of illnesses. For the gay and bisexual population, biosample research is particularly important for measuring the prevalence of human immunodeficiency virus (HIV). By determining people’s understandings of, and attitudes towards, the donation and use of biosamples, researchers can design studies to maximise acceptability and participation. In this study we examine gay and bisexual men’s attitudes towards donating biosamples for HIV research. Semi-structured telephone interviews were conducted with 46 gay and bisexual men aged between 18 and 63 recruited in commercial gay scene venues in two Scottish cities. Interview transcripts were analysed thematically using the framework approach. Most men interviewed seemed to have given little prior consideration to the issues. Participants were largely supportive of donating tissue for medical research purposes, and often favourable towards samples being stored, reused and shared. Support was often conditional, with common concerns related to: informed consent; the protection of anonymity and confidentiality; the right to withdraw from research; and ownership of samples. Many participants were in favour of the storage and reuse of samples, but expressed concerns related to data security and potential misuse of samples, particularly by commercial organisations. The sensitivity of tissue collection varied between tissue types and collection contexts. Blood, urine, semen and bowel tissue were commonly identified as sensitive, and donating saliva and as unlikely to cause discomfort. To our knowledge, this is the first in-depth study of gay and bisexual men’s attitudes towards donating biosamples for HIV research. While most men in this study were supportive of donating tissue for research, some clear areas of concern were identified. We suggest that these minority concerns should be accounted for to develop inclusive, evidence-informed research protocols that balance collective benefits with individual concerns.

## Introduction

As human biological samples (biosamples), including tissues such as such as blood or saliva, are used for an increasingly wide variety of diagnostic, treatment and research purposes, the ethical and legal issues surrounding them are becoming more complex, and are subject to increased public interest [1]. Tissues collected in healthcare and research settings are increasingly archived in biobanks [2] for potential use in future studies. Biobanks can be valuable for establishing risk factors and developing treatments, but the storage and use of biological material from donors raises practical and ethical issues around ownership, confidentiality, consent and feedback [1, 3–7]. The Nuffield Council on Bioethics summarise the balance required: *‘On the one hand*, *there is the view that the use of human tissue clinically*, *and for medical research*, *leads to benefits in diagnosis and treatment*, *and should be encouraged*. *On the other hand*, *there is concern to safeguard the individuals from whom tissue comes*, *and to ensure that tissue is used for acceptable purposes’* (p.2) [1].

The Gay Men’s Sexual Health (GMSH) survey is conducted with gay and bisexual men at commercial gay scene venues including bars, nightclubs and saunas in three Scottish cities: Glasgow, Edinburgh and Dundee. The survey has been conducted every three years since 1996, and has examined changes in gay men’s behaviour over that period. In 2005, the collection of anonymous oral fluid samples was added to the survey to complement the self-completion questionnaire. Each sample is linked to its corresponding questionnaire by barcode, but, to protect participants’ anonymity, neither element can be linked back to the participant. The saliva collected is tested for the presence of HIV antibodies to assess the prevalence of HIV, and linking samples with questionnaires allows for each participant’s actual HIV status to be compared with their self-reported HIV status. Crucially, the saliva sample is not collected for diagnostic purposes, and participants receive no feedback about their HIV status; the sample is used to measure prevalence within a population for epidemiological surveillance purposes. Participants are given information about accessing testing and counselling services.

The biosample collection component of the GMSH survey raises specific practical and ethical concerns related to participation and feedback. More generally, the collection of biosamples for research raises issues about informed consent, withdrawal from research, ownership of samples, and the destruction, storage and sharing of samples. While informed consent is clearly valued by research participants [8–12], the application of consent to secondary uses of donated biosamples is not straightforward [10]. For example a Finnish survey found that 30% of respondents felt that specific consent should be sought for each new use of a sample, 42% if the new study involved procedures that differ from in the original use and 58% if personal data are used in the research, while 34% would put no restrictions on research [12]. Lewis and colleagues [8] found that focus group participants tended to prefer generic consent when donating residual tissue for research. In a systematic review of qualitative research, Hill and colleagues found no consensus about an ideal model of consent [10]. Survey data from the UK indicates that increased understandings of, and opportunities to engage in discussion about, research are associated with acceptance of less restrictive models of consent [8].

Despite the dominant belief of research authorities that informed consent is essential to most research [1], evidence indicates that prospective donors pay relatively little attention to the written information provided to them during the process [13], that they rarely fully understand what they are consenting to [14], and that consent forms can be either oversimplified [15] or demand unrealistically high reading levels [16]. Busby [15] suggests that potential donors are vulnerable to being coerced by exaggerated descriptions of the value of their contributions. Furthermore, there is evidence that opt-in consent is a source of selection bias [9, 17–20]. While these are not arguments against the use of informed consent, they suggest that informed consent alone does not protect research participants from exploitation. Wendler and Emanuel [21] used survey data to build a tentative model of consent for research using stored, donated biosamples, in which consent should be obtained for research using identifiable samples collected for clinical use, but should not be necessary for the use of anonymised samples originally collected for research purposes. Hansson and colleagues argue that specific consent for secondary uses of biological samples is not necessary as long as a mechanism for withdrawal is in place [22]. Like consent, the parameters of withdrawal are not necessarily straightforward; for example Eriksson and Hegelsson argue that the onus should be on participants who wish to withdraw to justify their reasons for withdrawal, and that those reasons must not be based on misconceptions [23].

Ownership of donated biosamples is another issue raised by research using biosamples [7, 24, 25]. While the MRC advise that donations should be part of a ‘gift relationship’ that reduces ‘uncertainty over ownership’ [26], Dixon-Woods and colleagues [27] argue that ethical policy based on Titmuss’ model of altruism without expectations of reciprocity [5], is unsuitable for modern medical research, in which distinctions between not-for-profit and commercial research are not always distinct. A lack of consensus about ownership impacts decisions about whether biosamples should be destroyed after initial use or stored for future uses, and whether those stored samples should be shared with other organisations.

One obstacle to achieving balance between making effective use of biosamples in research and protecting donors’ is a lack of public awareness of research involving biosamples [28]. Despite the rapid growth of biobanking [2], public knowledge and understandings are limited; 91% of respondents to a large Finnish survey had either never heard of biobanking or had heard it but did not know what it meant [12]. As such, researchers studying public attitudes may find that those attitudes are not well-developed. However, examining public perceptions of the issues surrounding biosample donation is vital if researchers are to design and conduct research that leverages the various benefits of using human biosamples for research while protecting the donors of those biosamples.

To explore public understandings of, and attitudes towards, donating biosamples for research, we interviewed men from the same population as the GMSH survey. This paper focuses on findings from discussions about ethical and practical issues involved in donating biosamples for research, and the storage and reuse of biosamples. To our knowledge this is the first study of gay and bisexual men’s attitudes towards donating biosamples for research, and as such it will contribute to an extensive, and growing, base of evidence base about public perceptions of biosample donation. While the ethics of biosample donation are important for any target population, understanding the specific experiences and beliefs of gay and bisexual men is valuable for the development of research methods and guidelines tailored to the specific needs of that community, the member of which may have a heightened familiarity with participating in research, including that carried out in nightlife venues, as well as heightened sensitivities around blood-borne viruses.

## Methods

Ethics approval for the study was obtained from the ethics committee of the University of Glasgow, College of Social Science.

### Sample and recruitment

Semi-structured telephone interviews were conducted in August and September 2011 with 46 men aged between 18 and 63 (Table 1). Participants were recruited in thirteen bars (seven in Edinburgh, six in Glasgow) that were also used as data collection sites for the 2011 GMSH survey [29]. A sampling frame of bars diverse in terms of size and the demographics of their regular clientele was chosen to ensure a cross-sectional sample. Each venue was visited twice by a fieldwork team: once between 19:00 and 21:00 and once between 21:00 and 23:00. Each visit lasted 30 minutes. The fieldwork teams each comprised five fieldworkers and one team leader. During each visit, the fieldworkers approached every man in the venue to describe the research and, if they identified as men who have sex with men, invited them to participate in a telephone interview. Fieldworkers asked each man whether they took part in the 2011 GMSH survey and, if so, whether they provided an oral fluid sample as part of the survey. Each man was provided with an information sheet describing the interview process and the purpose of the research, explaining their right to withdraw, ensuring that their confidentiality would be protected and providing contact information for the principle investigator and local sexual health services. Upon agreeing to participate in a telephone interview, fieldworkers obtained each participant’s written, informed consent, first name and a contact telephone number, and agreed a date and time for the interview. Fieldworkers aimed to recruit a minimum of 40 participants, and ultimately recruited a total of 46, which was ajudged to be sufficient to capture a broad range of experiences and perspectives. Interviews were conducted with each of the 46 respondents. Of the 46 participants, 23 were recruited in Glasgow and 23 in Edinburgh. Nineteen had completed the GMSH questionnaire and provided a saliva sample, 10 had completed the questionnaire but not provided a sample, and 17 had not taken part in the GMSH survey. Details of participants are listed in Table 1.

**Table 1:** Table of participants.

Pseudonym	Age	Recruitment Location	Participated in GMSH survey	Provided saliva sample for GMSH survey
Ben	45+	Edinburgh	Yes	Yes
Blair	25–34	Glasgow	Yes	Yes
Callum	25–34	Glasgow	Yes	Yes
David	25–34	Glasgow	Yes	Yes
Derek	Unknown	Glasgow	Yes	Yes
Hamish	35–44	Edinburgh	Yes	Yes
Hector	45+	Glasgow	Yes	Yes
Henry	45+	Glasgow	Yes	Yes
Keith	18–24	Glasgow	Yes	Yes
Kennedy	35–44	Edinburgh	Yes	Yes
Lewis	18–24	Edinburgh	Yes	Yes
Nathan	Unknown	Glasgow	Yes	Yes
Norman	45+	Edinburgh	Yes	Yes
Ollie	25–34	Edinburgh	Yes	Yes
Roland	35–44	Edinburgh	Yes	Yes
Ross	18–24	Edinburgh	Yes	Yes
Simon	25–34	Glasgow	Yes	Yes
Taylor	45+	Glasgow	Yes	Yes
Tomas	18–24	Edinburgh	Yes	Yes
Brodie	35–44	Edinburgh	Yes	No
Bruce	18–24	Glasgow	Yes	No
Cameron	25–34	Glasgow	Yes	No
Daniel	25–34	Glasgow	Yes	No
Edgar	25–34	Glasgow	Yes	No
Edward	18–24	Glasgow	Yes	No
Harry	Unknown	Edinburgh	Yes	No
Homer	35–44	Glasgow	Yes	No
Kiram	45+	Edinburgh	Yes	No
Roger	35–44	Edinburgh	Yes	No
Bernard	18–24	Edinburgh	No	No
Carrie	25–34	Glasgow	No	No
Frank	25–34	Edinburgh	No	No
Hugh	45+	Edinburgh	No	No
Jimmy	35–44	Glasgow	No	No
Kelvin	18–24	Edinburgh	No	No
Kenneth	45+	Edinburgh	No	No
Kevin	45+	Glasgow	No	No
Kyle	45+	Edinburgh	No	No
Nic	25–34	Glasgow	No	No
Raphael	18–24	Glasgow	No	No
Ronald	35–44	Edinburgh	No	No
Sean	24–34	Edinburgh	No	No
Stanley	45+	Glasgow	No	No
Toby	24–34	Glasgow	No	No
Trevor	45+	Edinburgh	No	No
Yestin	Unknown	Glasgow	No	No

### Interviews

Interviews were carried out by telephone by the same fieldworker that initially recruited the participant. Each interview lasted approximately 30 minutes, and interviewees received a £20 gift voucher for their participation. The interview schedule contained questions about four topics: the GMSH survey; receiving individual feedback or test results from research; participation in different types of research studies; and donating biosamples. This paper comprises an analysis of the responses to questions about donating biosamples, which included open-ended questions about: informed consent; anonymity and confidentiality; ownership of samples; storing samples; sharing samples with other organisations; and perceptions of the varying sensitivity of different types of biosample. Interviews were digitally recorded with participants’ permission.

### Analysis

The recorded interviews were transcribed verbatim, checked and anonymised. Transcripts were imported into NVivo 9 to enable systematic comparisons to be made across the large set of data. Data were coded and charted systematically, following the principles of framework analysis [30], based on a coding frame developed by LM, SH and Andressa Gadda. The coding frame comprised the following codes: barriers to donating biosamples; views on destroying tissue samples; ethical considerations; views about ethics committees; experiences of donating tissue samples; misunderstandings and uncertainties; motivation and encouragement; professionalism of biosample collection; perceptions of tissue samples as property; the right to withdraw; sensitivities of different tissue samples; views on sharing samples with other organisations; and storing and destroying tissue samples. The data were coded by LM, SH and Andressa Gadda, and the research team regularly reviewed coding to identify and incorporate emergent codes and resolve disagreements by reaching consensus. Relevant themes that emerged from the initial analysis included: the value of research in promoting prevention; fear of breaches of research ethics; time and convenience as barriers to research participation; biosamples as gifts, and donation as altruism; and lay knowledge of medical research. Data were rigorously examined and cross-compared by CP to identify typical quotes and common reasoning and ensure that the breadth of responses pertaining to each theme was captured and represented, with particular attention given to atypical cases [31].

## Results

The results are reported in four different sections: informed consent, anonymity and confidentiality; rights of ownership and withdrawal of donated biosamples; destruction and reuse of biosamples; sharing biosamples after initial use; and sensitivities of donating different types of biosamples. These sections are based on themes that emerged during the coding process as key areas of discussion.

### Informed consent, anonymity and confidentiality

Participants typically valued being informed about research aims and the uses of donated biosamples. Jimmy, who had not taken part in the GMSH survey, believed participants should be *‘told exactly how it will be done*, *what the purposes of it are for and so the person knows that*, *yeah*, *this is really worthwhile doing this*, *and I’ve been told exactly what’s happening’*. Some valued knowing what will happen to samples following their initial research application; Ollie wondered *‘what will happen to them once the research is over–are they going to be sold or are they going to be destroyed–and what will happen with the information that’s gathered as a result of collecting the sample*?*’*. Most participants suggested that informed consent, either generic or specific, must be given. Nathan preferred specific consent:

*‘I’d rather know where it was going and what it was being used for than*, *like*, *not being sure–unless they would contact you to tell you*, *“You know*, *right*, *this is what’s happening*,*” then I would rather know there and then where my sample’s going.[…] I know that I would feel uncomfortable knowing that*, *if I asked where it was going and they go*, *“Oh*, *we can’t tell you*,*” and I wouldn’t do it then*. *Automatically*, *I’d rather know what was happening with it*, *first*.*’—*Nathan

Participants typically valued anonymity and confidentiality. Ross regarded confidentiality as particularly important when samples could indicate HIV status, which could have *‘insurance repercussions’*.

### Rights of ownership and withdrawal of donated biosamples

Many participants felt that donated biosamples should remain property of the donor, potentially ceasing at death, and participants often spoke about samples using terms implying ownership such as *‘my sample’*. Kennedy characterised samples as *‘your property being used elsewhere’*.

Ownership was often linked with samples’ origins within their donors’ bodies, or being *‘part of their DNA’* (Stanley). A small group of participants indicated that a sample’s bodily origin complicates the prospect of it being stored and shared with other research organisations; Nathan suggested that it is *‘a bit weird’* to keep a sample, which is *‘part of yourself’*, in storage. Bruce argued that donors should retain control of decision-making relating to their sample because *‘it’s always their property and their body’*. Harry equated sharing samples with private companies with *‘selling a part of you’*. Lewis identified that *‘it seems weird to kind of be so attached to your skin cells’*, but nonetheless felt that donors should determine how *‘your cells and your DNA’* are used. Some participants exhibited uncertainty about ownership (S1 Box). David drew a distinction between biological and moral interpretations of biosample donation: in the former, he saw donated biosamples as sufficiently disassociated from the individual that is cannot be their property, while in the latter he perceived samples as a permanent part of the donor.

The right to be informed about secondary uses of samples was linked with the concept of ownership. Stanley typified that perspective: *‘it’s still their sample*, *so if there’s going to be other tests done on it*, *I think they should be informed’*. Some participants mentioned the concept of a time period, varying between two and five years, after which the donor may cede control of a sample.

Some participants suggested that donors cease to own biosamples upon donation. Ben likened donation to spending money on a gambling machine: *‘Well*, *once you’ve donated it*, *you’ve donated it*. *It’s like putting money in the bandit*. *It’s your money*, *but once it goes through the slot*, *it’s the pub’s money’*. Derek likened donation with having a photograph taken: *‘it is a bit like when your image is captured on a camera*, *it no longer belongs to you–it*, *you know*, *belongs to someone else’*. Some described donated samples as gifts; Harry stated that *‘It’s a gift*. *There you go*. *If I donate a bit of my tissue*, *a bit of my hair or whatever*, *saliva*, *then I’m quite sure it’s yours to do what you want to do with it’*.

Many acknowledged that rights of ownership and control are determined at the point of giving consent:

*‘it all depends on what you were told when you gave the sample and what you agreed to when you gave the sample*. *So*, *obviously*, *if you’ve agreed that it can be used*, *stored and used for other purposes*, *and you’ve agreed that an ethical committee can decide*, *then you’ve signed over your rights of any further claim on that material*.*’*–Kenneth

Most participants believed donors should be able to withdraw from research and request that stored samples be destroyed. Withdrawal was identified as a human right essential to *‘any good research study’* (Ollie). Ross suggested that, while the right to withdraw provides comfort, he doubted many would exercise it. Keith agreed that withdrawal rights are important, but suggested researchers should *‘constantly remind people’* of potential benefits of research to discourage withdrawal. Derek perceived the withdrawal process as inherently opaque, meaning that those who withdraw must blindly trust that their sample will be destroyed.

Some participants did not regard withdrawal as an essential right, perceiving relinquishing control to be an implicit part of donation. Hamish stated that withdrawal is unnecessary if samples are anonymised.

### Destruction and reuse of biosamples

Participants typically indicated that biosamples should be destroyed after initial use, preventing non-consensual use, ensuring confidentiality and preventing undesirable organisations from acquiring samples. Some suggested prospective donors would be *‘nervy’* (Daniel) or *‘suspicious and paranoid’* (Hugh) about stored samples being misused or falling into *‘the wrong hands’*, such as commercial companies. Roland stated that *‘nobody really wants their*, *you know*, *sample floating around*, *you know*, *with DNA on them and whatnot’*. David suggested samples could *‘be used for some sort of nefarious scheme to*, *I don’t know*, *monitor you*, *clone you*, *steal your identity*, *all that sort of thing’*. Trevor had concerns about abuse, but expected that safeguards would protect against it: *‘I would like to think*, *anyway*, *that a system that was put in place would be very much protected’*.

Participants speculated about various practical issues that could arise when samples are not destroyed, including: the need to store contaminated samples securely; threats to anonymity and confidentiality; the resource costs of storing samples; the degradation and contamination of samples over time; and the diminishing relevance of samples over time.

Several participants expressed ambivalence towards destruction of samples. Norman stated that: *‘It doesn’t really bother me*, *you know*? *You’ve given the sample*, *so if it’s used once and destroyed or it’s used ten times and then destroyed*, *it’s really no skin off my nose because I’ve already given it’*. Hamish suggested that being unaware of secondary uses of samples is preferable: *‘as long as [*..*] it wasn’t linked back to me’*. Jimmy initially stated that he was not concerned, before questioning himself: ‘*[It] doesn’t bother me if they were destroyed*, *because you know no-one at all is going to find out if they’re destroyed*. *Even though you don’t know*, *you know*, *something could happen where these results come out and you’re like*, *“Oh no*,*” you know*?*’*.

Many participants’ responses to questions about destruction and reuse of samples suggested they had given little consideration to the issue prior to the interviews, and were forming initial opinions during the interview process, as Roland’s answers exemplify (S2 Box). Three participants explained that they could not form opinions on the merits of destruction without more specific information about intended uses of stored samples and the feasibility of secondary uses.

The most commonly mentioned disadvantage of destroying samples was that it precludes their use in further research, which was portrayed as wasteful, while storing samples for reuse was characterised as an opportunity to extract more value from each sample and avoid inconveniencing the public with further data collection. Many supported the storage and reuse of samples with caveats, including: the sample must be stored safely; further uses must be for worthwhile causes; the secondary use is similar to the research for which the sample was originally donated; the donor maintains the opportunity to withdraw; the donor is notified about uses of their sample; consent is given; and anonymity is protected.

Some participants suggested that reuse of samples might help to protect donors’ anonymity; Ollie stated that the use of *‘recycled’* samples is *‘probably more anonymous’*, perhaps implying that a lower number of donations reduces the risk of breaches of anonymity, or that anonymity is likely to be protected in a biobank setting.

### Sharing biosamples after initial use

Many participants favoured sharing biosamples with other research organisations, with some conditions. Some suggested that acceptability of sharing depends on the type of research being undertaken, with some characterising non-medical purposes as unacceptable. Medical benefits were typically portrayed positively, and commercial interests negatively. Roger explained that sharing is acceptable if the organisations using the biosample *‘are trying to make medicines*, *you know*, *that will help people’*. Some participants perceived commercial companies as having lower data security standards, being less trustworthy and being *‘all about money these days’* (Toby). Some participants opposed sharing of biosamples, citing concerns about breaches of anonymity, confidentiality and data protection, ‘misuse’, and biosamples being sold for profit.

### Sensitivities of donating different types of biosamples

Participants were asked about their perceptions of the sensitivities associated with donating different types of biosample. Many perceived no type as more sensitive than others, but some identified differences. Frank stated: *‘It’s one thing giving a swab of saliva*, *you know*? *But if you’re giving*, *you know*, *clippings of your hair or bits of your semen or blood*, *you know*, *if you’re having a blood test done*, *that can be quite private’*. Many identified blood as sensitive. Norman stated: *‘Giving blood*, *I nearly faint*, *so I mean*, *that’s not a good one for me*. *Saliva*, *no problem*, *urine*, *no problem*, *but blood*, *not a very good one for me’*. Nic suggested that blood is more sensitive than other tissues because of its preciousness, the information it can contain and its role in illness:

*‘you can read a lot more from blood than what you could from*, *say*, *a toenail*. *[*…*] I think that blood is pretty precious to everyone*, *and that not everyone likes getting blood taken from them*. *But as well as that*, *you know*, *there is a lot of blood-borne viruses*, *there is a lot of blood caught*, *no*, *I wouldn’t say blood caught–but there’s a lot of things that blood does*, *as opposed to*, *say*, *I don’t know*, *a flake of hair or a toenail’*.*—*Nic

Some participants perceived sensitivity as contingent on the location of tissue within the body. Lewis perceived internal tissues as more sensitive than external tissues, while Nathan implied that tissue located in the anus or bowels is sensitive: *‘Anything from*, *you know the back of you*, *I think might be a little bit personal’*. Some associated sensitivity with intimacy; urine, semen and bowel tissue were characterised as intimate, and saliva, hair, skin and fingernails as less intimate.

Some participants indicated that sensitivity depends on the collection environment; saliva was identified as straightforward to donate in a non-clinical, informal setting, such as a bar, while hair and urine were identified as less comfortable to donate in such a setting. Keith stated: *‘If you asked me to give a semen sample in the middle of the pub*, *I’d be a bit offended’*.

For some, sensitivity depended on the collection method. Tomas perceived hypodermic needles as discouraging: *‘I’ve got a thing with needles*, *so I think anything that involved kind of ‘needley’ things*, *that’s when I would go*, *“Ooh*, *ok*.*” That’s where I would probably not be able to do it–but telephone interviews*, *swabs and stuff*, *that’s fine’*.

Perceptions of the sensitivity of different types of biosamples were not consistent between participants. Opinions about the sensitivity of donating urine and hair biosamples were varied. Some identified blood as the most sensitive tissue, while others perceived semen as most sensitive. Different types of sensitivity were mentioned; while donating blood was often associated with the pain and fear of hypodermic needles, donating semen was associated with *‘embarrassment’* (Simon), situational inappropriateness and its reproductive capacity; Tomas stated that: *‘I kind of worry that it would end up*, *I don’t know*, *getting used and I’d end up with a child in nine months or something’*.

## Discussion

The participants presented diverse views, offering a range of interpretations of issues including the ownership of samples and the relative sensitivities of different types of tissue. Participants presented a high degree of agreement on some issues, including the value of informed consent and other key elements of research ethics and wariness about the involvement of commercial organisations in biosample donation. Participants sometimes contradicted themselves, and some appeared not to have considered the issues discussed prior to the interviews. Those participants may have been forming initial opinions during the interviews, which may be symptomatic of low public awareness of the concept of biosample donation and the issues surrounding it.

Participants predominantly valued informed consent, security, anonymity and the right to withdraw from research. The perceived importance of consent echoed Lewis and colleagues’ [8] quantitative findings. Many perceived biosamples as the continuing property of the donor, echoing findings of Waldby and colleagues [4], and typically derived that ownership from the biosample’s origin within the body. As found by Datta, Wellings and Kessel [9], our participants identified DNA as a marker of ownership. As McGuire observes, DNA is uniquely identifiable [32], and, unlike social identity, ‘bio-identity’ cannot be protected through anonymity [9]. One participant evoked Titmuss [5] by describing the biosample as simultaneously owned and not owned by the donor. Some participants exhibited little sense of attachment to bodily tissue, and some suggested that ownership ends at the point of donation. Datta, Welling and Kessel [9] found a similar range of perspectives regarding personal attachment to, and ownership of, blood. Widespread concern and diversity of opinions about ownership may highlight the need for the issue of ownership to be carefully addressed in participant consent forms.

Participants predominantly supported the destruction of biosamples following initial use, though some favoured storing biosamples for reuse. Some of the enthusiasm for destruction may have stemmed from a lack of awareness of the potential value of secondary applications, suggesting that biosample collection may benefit from providing potential donors with information about the nature and benefits of secondary uses. Many supported the sharing of biosamples with other organisations after use, but often with conditions about the nature of the organisation and the types of uses to which samples will be put, and with concerns about anonymity and misuse. Commercial organisations were generally viewed negatively, while advancements in medical science were viewed favourably, echoing existing research [10, 24, 33]. Lewis and colleagues [8] identified similar aversion to commercial organisations, as well as some misgivings about animal research and research conducted outside of the UK.

Some participants perceived differences in the sensitivity of donating different types of tissue. Blood, urine, semen and bowel tissue were commonly identified as sensitive, and saliva donation as relatively straightforward, echoing literature suggesting that saliva is largely a simple and non-invasive form of research data [24, 34, 35], particularly among male participants [36], and a more convenient diagnostic fluid than blood or urine [24, 37]. Conceptions of saliva differ greatly from blood, which, as Datta, Welling and Kessel suggest, is imbued with complex cultural significance, embodying various contradictory identities, while saliva is generally conceived as relatively straightforward waste material [9]. Waldby and colleagues suggest that attitudes towards sharing blood can depend on the risk that individual donors attach to their blood [4]; conceivably, this could also apply to potential donors with saliva-borne diseases. As such, researchers seeking to collect biosamples may benefit from considering the particular significance that their target population may attach to the type of tissue to be collected.

Secko and colleagues argue that *‘public consultation related to biobanks has been largely oriented to assuring and informing rather than seeking considered input’* (p.781). In 1995, Macintyre [38] urged scientists to base genetic research policy on evidence about public attitudes and behaviours, rather than assumptions, suggesting that by doing so science can achieve balance between collective benefits and legitimate individual concerns. Education about research methods and procedures may be effective in increasing public acceptance of donating biosamples for research [10, 24], but improving ethical research practice should be a collaborative process, not conciliatory one [28]. As Macintyre suggests, *‘attempts to improve the public understanding of science should be complemented by attempts to improve the scientific understanding of the public’* (p.231) [38].

While understandings of public attitudes towards biosample donation derived from quantitative data, such as the model of consent tentatively proposed by Wendler and Emanuel [21], are likely to be effective from a utilitarian perspective, it is important to acknowledge the legitimacy of the less common ethical concerns. Much of the value of qualitative research lies in highlighting atypical perspectives, and recognising their legitimacy [31]. Our findings echo existing evidence suggesting that the public are predominantly well-disposed towards participation in research involving biosamples [24, 27, 39], but that supportiveness was often mediated by concerns and caveats, and engaging with these issues is part of achieving robust ethical research policy.

A limitation of the research design is the use of hypothetical questions to predict behaviours, as people do not necessarily behave the way that they say they will. Further, these findings are difficult to generalise because ethical issues likely differ substantially between different social contexts, and our participants were taking part within a gay men’s health context. Taking these limitations into account, this research contributes to the wider body of evidence around public attitudes to biosample donation and biobanking.

To our knowledge this is the first study of attitudes to donating biosamples that focuses on gay and bisexual men, a group for which the practicalities and ethics of biosample donation are of heightened importance to gay and bisexual communities due to specific and elevated sensitivities. Our analysis provides a nuanced and novel insight into participants’ understandings of, and attitudes towards, donating biosamples for research in a gay men’s health context. Regarding specific types of tissues, our research adds to the body of literature highlighting the value of saliva as a particularly convenient diagnostic fluid [24, 34, 35]. Our conclusions should be considered within the existing body of evidence when developing research methods and ethical guidelines.

## Conclusions

This study of gay and bisexual men’s attitudes towards donating biosamples for research purposes suggests that potential donors have concerns pertaining to the donation, storage and reuse of biosamples, largely focused on issues of: confidentiality and anonymity; informed consent; the right to withdraw; ownership of biosamples; data security; and potential misuse, particularly by commercial organisations. We suggest that, regardless of evidence of widespread support for donating biosamples for research, these concerns should be acknowledged in the interests of developing ethical practices for biological and genetic research that strike a balance ‘*between self and society’* (p.903)[9]. If public interests are largely aligned with those of medical research, then there is reason to believe that that reaching a balance between individual and collective concerns need not be a struggle.

## Supporting Information

S1 Box Uncertainty about ownership of donated biosamples. (DOCX)

S2 Box Forming opinions about destruction and reuse of biosamples. (DOCX)
