# Supplementary material for: Gay and Bisexual Men’s Perceptions of the Donation and Use of Human Biological Samples for Research: A Qualitative Study
Source: PLoS One. 2015 Jun 8;10(6):e0129924. doi: 10.1371/journal.pone.0129924 (PMC4459996; doi:10.1371/journal.pone.0129924)
Supplement: S1 Box — (DOCX) [file pone.0129924.s001.docx]

Supporting Infortmation Box 1: Uncertainty about ownership of donated biosamples

Interviewer: *Do you think there is a point, and if so, when would it be, that a person’s tissue sample is no longer practically theirs to make a decision over?*

Daniel: *As soon as it’s handed over. As soon as it’s handed over, it would become property of whoever, but with DNA and all the rest of it, it’s probably still, always, the person who gave it.*

Interviewer: *Do you think there is a point, and if so, when it would be, that a person’s tissue sample is no longer practically theirs to make decisions over?*

Frank: *No.*

Interviewer: *No?*

Frank: *There’s no point. There’s no point in time that, well, it wouldn’t be theirs, because – well, it depends what they want to do with it, or what they’re testing for, or if they’re going to, you know, it goes back to the purpose that you get it, I suppose. But no, to answer your question, no – at no point. I suppose then, someone else might say, well, as soon as you’ve kind of donated it, it stops becoming yours.* *But it’s just, I don’t know – I’m kind of going off on a tangent, but I suppose, ethically, no, it doesn’t stop being their sample.*

Cameron: *That’s a stuck down the middle because, on one hand, yeah, you’re relinquishing – you’re offering it up, you’re relinquishing it – but at the same time, it’s still part of who you are. It’s still part of something that you gave, so I don’t know, I’d need to sit on the fence with that one.*

Interviewer: *Do you think researchers should share samples with private or commercial companies?*

Nic: *No.*

Interviewer: *No?*

Nic: *Absolutely not. I agree to certain things, but I don’t agree to, say, my blood being sold off to, I don’t know, a cosmetic company to try out something on that, or a hair or whatever – I don’t agree to that.*

*[...]*

Interviewer: *Can you think of, you know, say you agree to give a tissue sample and you go to wherever it’s getting done and it’s taken and it’s taken away to a lab, and then the tests are done, when do you think the point is that, or do you think that, in that sort of process, is there a point where it’s no longer theirs, the person who donated the sample, to make the decisions over?*

Nic: *Well, I think that that was the agreement of giving the sample, anyway.*

Interviewer: *Ok, so kind of when it’s handed over?*

Nic: *Yeah, when it’s handed over, it’s not mine anymore, no. I would agree to that.*

Interviewer: *Yeah, do you think there is any point, and if so, when would it be, that a person’s tissue sample is no longer practically theirs to make decisions over?*

David: *In a biological sense, I would say that if it’s you know become so degraded in the sense that it’s broken down to coming to choose your own parts like you know, you’re looking at mitochondria separate from every other part of the cell or that sort of thing, that’s when it would stop, when it is broken down so much that there’s absolutely no associating each part with the rest of the cell that’s been blown apart sort of… but in a moral kind of sense, it is always part of the individual and you should always be responsible for your own… for yourself.*
