# Supplementary material for: Gay and Bisexual Men’s Perceptions of the Donation and Use of Human Biological Samples for Research: A Qualitative Study
Source: PLoS One. 2015 Jun 8;10(6):e0129924. doi: 10.1371/journal.pone.0129924 (PMC4459996; doi:10.1371/journal.pone.0129924)
Supplement: S2 Box — (DOCX) [file pone.0129924.s002.docx]

Supporting Information Box 2: Forming opinions about destruction and reuse of biosamples.

Interviewer: *When tissue samples are donated to research, they’re either stored to reuse for other research, or they are destroyed. What do you think are the advantages of storing tissue samples for reuse?*

Roland: *For reuse?*

Interviewer: *Yeah.*

Roland: *I don’t know. Would it be to, I don’t know, to test for other, or to use for other reasons other than sexual health? I don’t know, to be honest.*

Interviewer: *That’s a pretty good guess, that’s good yes. Again, you don’t have to know the answers to all these questions, but it’s just trying to elicit what you do know. So you’re not always going to know – you know, these questions, you don’t think about, do you?*

Roland: *No, no, you don’t. No, definitely not.*

Interviewer: *So we’re pulling your brain apart here, with some of these questions.*

Roland: *Yes.*

Interviewer: *So are there sort of disadvantages of storing tissue samples, do you think?*

*Disadvantages of storing them?*

Interviewer: *Yeah.*

Roland: *From the, disadvantages to the person who the samples comes from or disadvantages…*

Interviewer: *Well, initially, yes, to the donator – would there be any disadvantages for them, knowing that their tissue sample is being stored somewhere?*

Roland: *Yeah, I suppose the only disadvantage – well, I don’t know. I’d probably go back to the how secure the samples are and, you know, would the donator know exactly what they were being used for.*

Interviewer: *Ok. And what do you think are the benefits of destroying tissue samples after a single use?*

Roland: *After single use? I think the benefits to the person who donates it is they know, you know, they’re safe in the knowledge that what they’ve agreed for it to be used for is exactly that.*

Interviewer: *Right, ok. Do you think there are any pitfalls of destroying tissue samples after single use?*

Roland: *Previously, I would have said no, but now that you asked me that previous question about possibly using them for other things, then yes, there might be pitfalls for destroying it.*

Interviewer: *Ok.*

Roland: *But if you hadn’t asked me that before, I would have said no, if that helps.*
